# VO_2_ Thermochromic Films on Quartz Glass Substrate Grown by RF-Plasma-Assisted Oxide Molecular Beam Epitaxy

**DOI:** 10.3390/ma10030314

**Published:** 2017-03-19

**Authors:** Dong Zhang, Hong-Jun Sun, Min-Huan Wang, Li-Hua Miao, Hong-Zhu Liu, Yu-Zhi Zhang, Ji-Ming Bian

**Affiliations:** 1 New Energy Source Research Center, Shenyang Institute of Engineering, Shenyang 110136, China; ambitious211@163.com; 2 Key Laboratory of Materials Modification by Laser, Ion and Electron Beams(Ministry of Education), School of Physics and Optoelectronic Technology, Dalian University of Technology, Dalian 116024, China; jun8894@163.com (H.-J.S.); wmhkjt@mail.dlut.edu.cn (M.-H.W.); miaolihua@163.com (L.-H.M.); liuhz418@163.com (H.-Z.L.); 3 Key Laboratory of Inorganic Coating Materials, Chinese Academy of Sciences, Shanghai 200050, China; yzzhang@mail.sic.ac.cn

**Keywords:** metal-insulator transition, transition-metal oxides, vanadium dioxide (VO_2_), oxide molecular beam epitaxy

## Abstract

Vanadium dioxide (VO_2_) thermochromic thin films with various thicknesses were grown on quartz glass substrates by radio frequency (RF)-plasma assisted oxide molecular beam epitaxy (O-MBE). The crystal structure, morphology and chemical stoichiometry were investigated systemically by X-ray diffraction (XRD), atomic force microscopy (AFM), Raman spectroscopy and X-ray photoelectron spectroscopy (XPS) analyses. An excellent reversible metal-to-insulator transition (MIT) characteristics accompanied by an abrupt change in both electrical resistivity and optical infrared (IR) transmittance was observed from the optimized sample. Remarkably, the transition temperature (T_MIT_) deduced from the resistivity-temperature curve was reasonably consistent with that obtained from the temperature-dependent IR transmittance. Based on Raman measurement and XPS analyses, the observations were interpreted in terms of residual stresses and chemical stoichiometry. This achievement will be of great benefit for practical application of VO_2_-based smart windows.

## 1. Introduction

The continuously increasing energy consumption around the world is posing a problem not only to the environment, but also to human health. Especially buildings are said to be responsible for about 40 percent of the world’s total annual energy consumption owing to the excessive use of lighting, air conditioning and heating [1]. The increased use of a heating/cooling system in buildings to maintain comfortable environments has led to a meteoric rise in electricity utilization and carbon dioxide emissions during the electricity generation process [2]. Therefore, it was highly desirable to develop some alternative technologies for heating and air conditioning systems to combat the energy crisis. One of such alternatives is to construct ‘smart windows’ with thermochromic coatings, which can control the amount of solar radiation entering or black-body radiation leaving a building intelligently according to the ambient temperature [3,4,5,6]. By virtue of its unique and fascinating properties, VO_2_ was considered to be one of the key materials for a wide range of energy-related applications, which have been proven as the most promising thermochromic coating for smart window application [7,8]. It undergoes an abrupt reversible phase transition, known as metal-to-insulator transition (MIT) or semiconductor-to-metal (SMT) first-order transition. Most notably, this allotropic transition in crystal symmetry and electronic band structure, which can be triggered by ambient temperature stimuli, was usually accompanied by an abrupt and dramatic change in optical transmission especially within the infrared (IR) wavelength region [9,10,11,12]. At temperatures below the transition temperature (T_MIT_), VO_2_ is in the semiconducting state (M1 phase) with high transparency in both the visible and IR wavelength region, which allows most of the radiant solar energy to transmit into the VO_2_-coated windows; while at temperatures above TMIT, VO_2_ is in the metallic state (R phase) with high reflection in the IR region while keeping almost the same transparency in the visible region, which makes most of the IR solar radiation reflected by VO_2_-coated windows. Since IR solar radiation carried about 50% of the total solar energy, the intelligent adjustment of indoor temperature could be realized through the VO_2_-coated smart window without consuming extra energy; as a result, tremendous energy consumption for the heating/cooling system could be conserved [5]. However, it should be noted that the growth of high quality VO_2_-coated glass with high IR transmittance contrast was still rather challenging due to the complexity of the vanadium-oxygen system [9,10].

So far, various substrates have been employed for the growth of high quality VO_2_ thin films, such as sapphire (Al_2_O_3_), titanium dioxide (TiO_2_), magnesium fluoride (MgF_2_) and fused quartz glass [7,8,9,10]. The major advantage of the quartz glass substrate lies in its much lower cost, stable physical and chemical properties, as well as direct compatibility with smart windows. Moreover, many methods have been proposed and attempted to grow VO_2_ films since the MIT features of as-grown VO_2_ films are closely associated with the growth approaches, such as sputtering deposition, sol-gel, chemical vapor deposition (CVD), pulsed laser deposition (PLD) and oxide molecular beam epitaxy (O-MBE). Currently, the O-MBE technique has been extensively investigated as a suitable way for VO_2_ film epitaxial growth with well controlled chemical stoichiometry and controlled thickness on the atomic scale [9,10]. However, to the authors’ knowledge, there has been little investigation on the intimate correlation between structural, compositional and MIT properties of MBE-grown VO_2_ films on quartz glass, which may shed light on the mechanism responsible for the controllable MIT behavior in VO_2_ film.

In this study, VO_2_ thin films with a controlled thickness were grown on quartz glass substrates by radio frequency (RF)-plasma-assisted O-MBE. The crystal structure, morphology and chemical stoichiometry were investigated systemically by X-ray diffraction (XRD), atomic force microscopy (AFM), Raman spectroscopy and X-ray photoelectron spectroscopy (XPS) analyses. Especially the MIT behavior of VO_2_/glass samples was characterized by measuring the change magnitude in electrical resistance and IR transmittance during the heating and cooling process. The results indicated that the reversible MIT characteristics with narrow hysteresis width and large amplitude contrast in both IR optical transmittance and resistivity were realized for the optimized sample. Our achievement will be of great benefit for VO_2_-based smart window application.

## 2. Experimental Details

### 2.1. Thin Films’ Preparation

VO_2_ films were grown on quartz glass substrates by an RF-plasma-assisted O-MBE with a base pressure better than 5.5 × 10^−7^ Pa. Prior to deposition, a 20 × 20 mm^2^ quartz glass substrates were cleaned with a normal process to remove residual contaminants on its surfaces. A standard RF plasma source was used to provide reactive oxygen radicals with 6N O_2_ as the gas source controlled by a mass flow controller with a precision of 0.1 sccm (standard cubic centimeter per minute). The pure metallic vanadium powder (99.7% purity, Alfa Corp., Jiangsu, China) was evaporated by a customized e-beam evaporator system where the vanadium flux was controlled by a crystal oscillator (INFICON-SQM160, leyfond Technology Co., Ltd. In Shenzhen, Shenzhen, China). Details of the deposition process have been reported in our previous papers [10,13]. All of the parameters, such as substrate temperature, chamber pressure, and metallic vanadium evaporation rate, as well as the O_2_ flux rate, were optimized for high quality VO_2_ films. One of the advantages for the MBE technique was its excellent reproducibility, so we suppose that the film thickness can be well controlled in the current case. Three samples with controlled thickness were grown by adjusting the deposition duration time while keeping other parameters constant. The growth times were 10 min, 20 min and 40 min, and the corresponding film thicknesses were determined to be about 15, 30 and 60 nm, respectively. For convenience, the samples were labeled as S1 to S3 with increasing growth time.

### 2.2. Characterizations 

The crystallographic properties of the films were determined by X-ray diffraction (XRD) in regular θ–2θ scanning mode using a LabXRD-6000 (CuKa1: λ = 0.154056 nm, Shimadzu, Beijing, China). The diffraction photons were collected by the diffractometer from 10° to 80° with a 0.02° step size. The surface morphology was examined by atomic force microscopy (AFM) with tapping mode using MI PicoScan 2500 (Molecular Imaging, McDonough, GA, America). The MIT properties were investigated by monitoring the change in both electrical resistivity (by Keithley 2635A source meter, Keithley, Shenzhen, China) and IR transmittance (using an FT-IR FTIR SPECTROMETER, Bruker EQUINDX 55, Bruker, Bremen, Germany) across the MIT process, i.e., the samples were thermally cycled in the temperature ranging from 20 to 100 °C. The films composition and valence state of V were investigated by XPS analyses on Thermo Scientific ESCALAB250Xi system (Thermo Scientific, Waltham, MA, America) with an AlKα 1486.8eV X-ray radiation source under a base pressure of 3×10^−8^ Pa. Before measurement, the sample was sputtered by Ar ion bombardment for 30 seconds for surface cleaning. The O1s binding energy line at 530 eV was taken as a reference for calibration. Raman spectra measurements were carried out on a DXR Raman Microscope (Thermo Scientific) with a 532-nm excitation laser source.

## 3. Results and Discussion

### 3.1. Structural and Morphological Analysis

**Figure 1:**
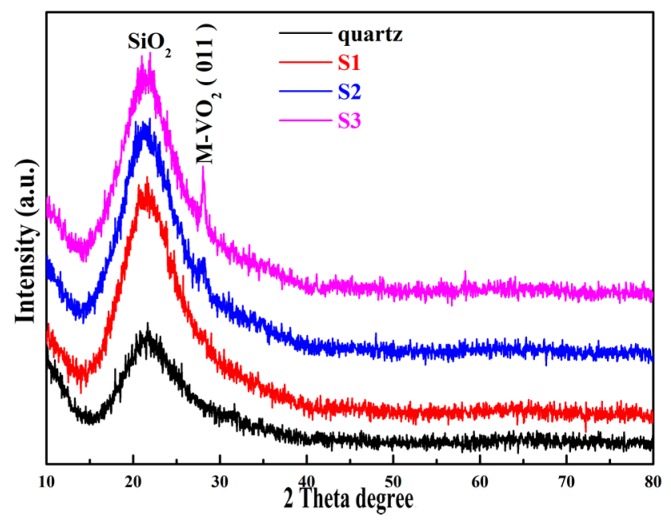
XRD patterns of VO_2_ films grown on quartz glass substrates by O-MBE with thickness controlled from S1 to S3.

Figure 1 shows the XRD patterns of the VO_2_ films grown on quartz glass substrates by MBE with controlled thickness. For comparison, the XRD patterns of the quartz substrate are also presented in the bottom of Figure 1. As can be seen from Figure 1, in addition to the dominant broad peak within 15° to 25°, which is due to the contribution of the amorphous quartz substrates, the well-defined sharp XRD pattern corresponding to the monoclinic VO_2_ (011) was observed at 28.04° for samples S2 and S3 (JCPDS 76-0675) [14]. No detectable characteristic diffraction peak can be observed from sample S1 probably due to its rather thin thickness. In addition, the gradual increases in the peak intensity and decrease in the full width at half maximum (FWHM) value of monoclinic VO_2_ (011) were observed with increasing thickness, indicating the gradually improved crystalline quality of as-grown VO_2_ film with increasing growth time. Most importantly, no clear diffraction peaks from other vanadium oxides (V_2_O_3_, V_2_O_5_, V_6_O_13_, etc.) were observed. The XRD results demonstrated that good monoclinic phase VO_2_ films with (011) preferred orientation have been obtained on quartz glass substrate by MBE.

**Figure 2:**
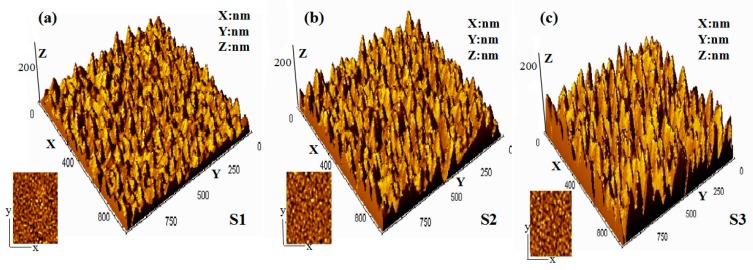
The surface morphology AFM 3D images (1 × 1 μm^2^) of VO_2_ films grown on quartz substrates by MBE with various thickness. (**a**) S1; (**b**) S2; (**c**) S3.

The surface morphology of the VO_2_ films with various thickness were characterized by AFM, and the AFM 3D images with the scanned area of 1 × 1 μm^2^ were shown in the Figure 2. From these images, all of the VO_2_ films exhibit a relatively flat surface with the root mean square (RMS) of 2.86 nm, 6.44 nm, 9.89 nm for samples S1, S2 and S3, respectively. This case was supposed to be resulting from the presence of tensile stress within VO_2_ film, as confirmed later by Raman spectra.

**Figure 3:**
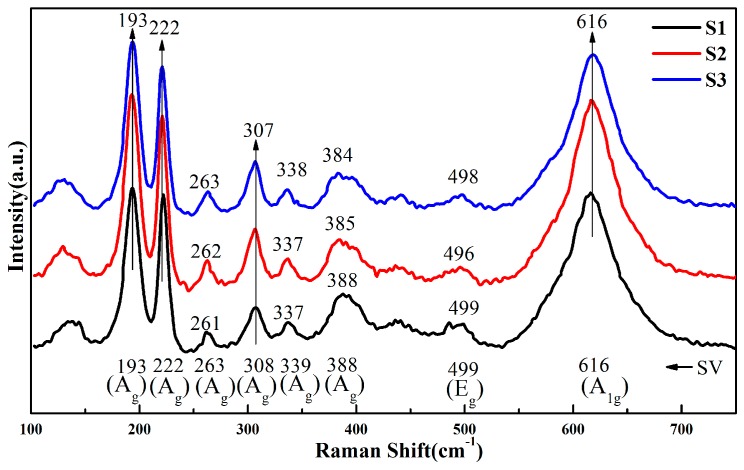
The Raman spectra for the VO_2_ films grown on quartz substrates by MBE with controlled thicknesses of 15, 30, and 60 nm for S1 to S3, respectively.

To investigate the internal stress within the VO_2_/glass samples, the Raman spectra measurements were carried out since the line shape of the Raman spectrum was extremely sensitive to the residual stress inside the films [15,16]. Figure 3 shows the Raman spectra for the VO_2_ films grown on quartz substrates by MBE with controlled thickness; the “SV” at the bottom denotes “standard value”. Here, the “standard value” refers to the value for single crystal VO_2_ free of stress. It can be seen from Figure 3 that all of the main peaks at 193, 222, 307 and 616 cm^−1^ can be well indexed to the monoclinic VO_2_, except the main peaks, where some tiny peaks and subtle peak deviations were also observed. Moreover, as the film thickness increased, the tiny peaks decreased, and every peak position substantially aligns well with the standard peaks of bulk VO_2_. Since the tiny peaks and peak deviation in Raman spectra were extremely sensitive to the residual stress within the films, thus these results indicated that the M-phase VO_2_ films were obtained on quartz glass substrate and the tensile stress gradually relaxed with increasing thickness, which was in good agreement with the XRD result. Given the remarkable difference in the thermal expansion coefficient between the VO_2_ films (1.71 × 10^−5^ K^−1^) and quartz glass substrates (5 × 10^−7^ K^−1^), the thermal tensile stresses can be expected in the as-grown VO_2_/glass structure. In addition, stresses originating from lattice mismatch between the VO_2_ films and quartz glass substrates are also supposed to be remarkable considering the fact that quartz is amorphous, while VO_2_ films are crystalline. Therefore, the Raman shift matches the total tensile stress. However, it is challenging to quantify thermal stresses from the current results. According to the [17], the lattice mismatch was supposed to be more likely responsible for the observed tensile stress [17].

### 3.2. Optical and Electrical Properties

**Figure 4:**
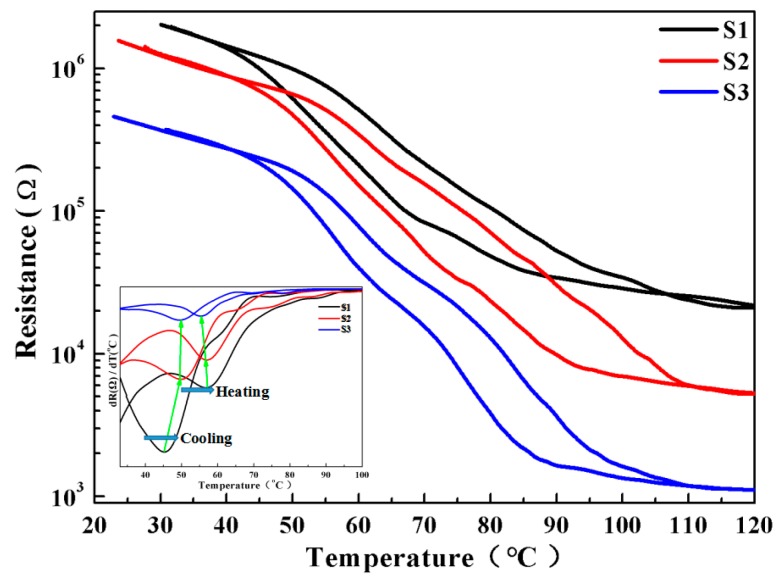
Thermal hysteresis loops of the sheet resistance of the VO_2_ thin films grown on the quartz substrates with controlled thicknesses of the VO_2_ over layer. The differential d(R)/d(T) versus temperature curves for heating and cooling branches are shown in the inset to determine the phase transition critical temperature (Tc) from the Gaussian fit of the peaks.

To study the MIT behavior of the VO_2_ thin films grown on the quartz substrate, the thermal hysteresis loops of sheet resistance during the heating and cooling process are recorded in Figure 4. The relatively smooth transition profiles were obtained from samples S1 to S3, suggesting a first-order reversible MIT phase transition behavior for all of the samples. The differential d(R)/d(T) versus temperature curves for heating and cooling branches are shown in the insets of Figure 4, and the Tc was determined from the Gaussian fit of the d(R)/d(T) curves (at which the value reaches its extremum). The MIT properties can be characterized by the following parameters: Tc (the MIT critical temperature), the hysteresis width (ΔH, defined as the difference of Tc for the heating and cooling branch), the transition sharpness (ΔT, characterized by the FWHM of the derivative curve of d(R)/d(T)-T plot), as well as the transition magnitude (amplitude, defined as the ratio of resistivity in the insulator phase to that in the metallic phase in the heating process). Herein, the detailed MIT parameters are summarized in Table 1.

A distinct MIT phase transition behavior with the transition amplitude of more than two orders of magnitude was achieved for our optimized samples, and the value of Tc (52.5 °C on average), ΔT (9.85 °C on average) and ΔH (8.6 °C on average) was in reasonable agreement with the reported value for crystalline stoichiometric VO_2_ films [9,10]. Moreover, as the film thickness increased from S1 to S3, the resistivity in both the insulator and metallic phase decreased remarkably, while the amplitude ΔA increased slightly (as shown in the inset of Figure 4). The dependence of the MIT properties of VO_2_ film on film thickness was supposed to be associated with tensile strain relaxation with increasing thickness; the detailed mechanism has been discussed and elucidated in our previous report [10,13]. What is more, it would also be related to the surface composition of the VO_2_ thin film, which would be confirmed later by XPS analysis.

**Figure 5:**
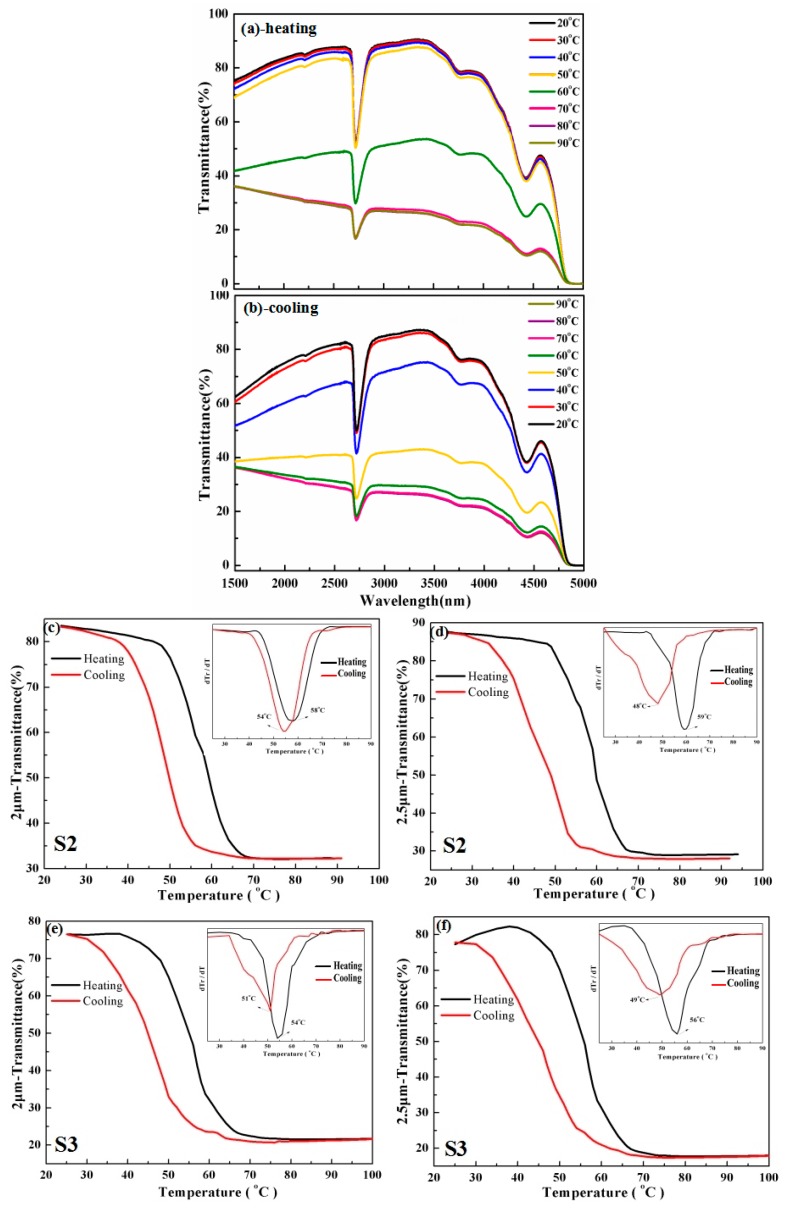
(**a**,**b**) The transmission spectrum of the VO_2_ sample grown on quartz substrate measured at various temperatures; (**c**–**f**) the hysteresis loop at 2.0 and 2.5 μm IR optical transmittance as a function of temperature for samples S2 and S3. The corresponding differential d(Tr)/d(T) versus temperature curves for heating and cooling branches are shown in the inset.

For the practical application of VO_2_-based smart thermal glazing of windows, the high contrast in IR optical transmittance between insulator and metallic state was highly desirable. Figure 5a,b shows the IR transmittance measured as a function of temperature across the MIT process. It should be noted that no reliable IR optical transmittance data were detected due to its rather thin thickness. As can be observed from Figure 5a,b, an obvious transmittance valley was observed at an ~2700-nm wavelength, which was supposed to be resulting from the proximity of the intrinsic absorption limit of the quartz glass substrate [18,19]. Moreover, as the temperature increased, the overall transmission decreased remarkably, which was related to the MIT features of VO_2_ film. Figure 5c–f shows the hysteresis loops at 2.0- and 2.5-μm transmission, which were obtained from the optical transmittance of samples S2 and S3 as a function of temperature; the differential d(Tr)/d(T) versus temperature curves for heating and cooling branches are shown in the insets of Figure 5c–f, and the Tc was determined from the Gaussian fit of the d(Tr)/d(T) curves (at which the value reaches its extremum). The MIT properties can also be characterized by several parameters just like those investigated above by the resistivity-temperature curve. Additionally, the detailed MIT parameters obtained by temperature-dependent IR transmission are summarized in Table 2. As the film thickness increased from S2 to S3, the value of Tc and ΔH decreased slightly just as the trend for the resistivity-temperature curve. Moreover, the rather high contrast of nearly three-fold was achieved in IR optical transmittance between the insulator and metallic state, which will be especially favorable for practical application of VO_2_-based smart thermal glazing of windows. The thermal hysteresis might be detrimental for certain applications, such as optical switching. For the thermochromic smart window application, a rather small hysteresis width will be especially favorable for rapid response to environmental temperature. Remarkably, the TMIT deduced from the resistivity-temperature curve was reasonably consistent with that obtained from the temperature-dependent IR transmittance.

### 3.3. XPS Composition Analysis

**Figure 6:**
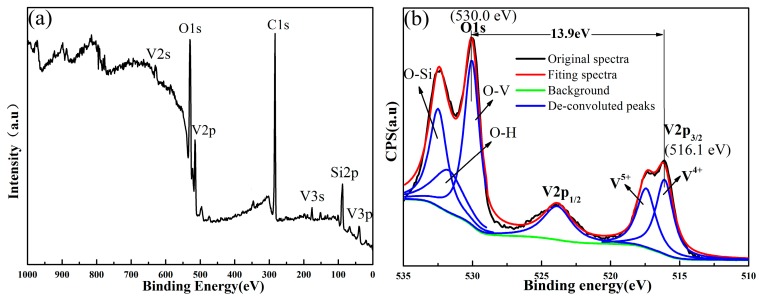
(**a**) XPS survey spectra with binding energy in the range of 0 to 1000 eV for sample S2; (**b**) the enlarged high-resolution spectra with binding energy in the range of 510 to 535 eV and the fitting results for sample S2. After subtracting the Shirley background (green curve), the V2p3/2 peak could be deconvoluted into V^4+^ and V^5+^ peaks.

Because of the complexity of the vanadium-oxygen system, a number of vanadium oxides (such as VO, V_2_O_3_, VO_2_ and V_2_O_5_) are prone to be formed with remarkable discrepancy in phase transition behavior [9,10]. Thus, precise control of the valence states of V in the VO_2_ layer was essential to achieve stable device performance for the samples. To be able to identify the valence state of V in the VO_2_ films grown on the quartz substrates, the optimized sample S2 was investigated by XPS, and the results are shown in Figure 6. The XPS survey spectra with binding energy in the range of 0 to 1100 eV is shown in Figure 6a; all of the elements can be well identified in the survey spectra according to previous reports in the literature [20]. The signals from C1s were detected for the sample, which could be attributed to unintentional contamination from the ambient environment during the growth and measurement process, since the C element was a ubiquitous element in the environment. To get the precise valence state of V in the oxide layer, the enlarged high-resolution XPS spectra focusing on O1s and V2p3/2 with binding energy between 510 and 535 eV are shown in Figure 6b. The O1s peak could be convoluted into two peaks; only the one at 530.0 eV was related to vanadium oxides, whereas the other peak at 532.5 eV comes from hydroxide and/or carbonate contamination [20]. The V2p3/2 peak, however, shows a shoulder on the high binding energy side, indicating that some surface oxidation has likely occurred in the sample film [21]. Meanwhile, the typical two-peak structure (V2p3/2 at 516.1 eV and V2p1/2 at 523.9 eV) due to the spin-orbit splitting was observed for the V2p signal. Additionally, the binding energy span was calculated to be 13.90 eV from Figure 6b. Here, the O1s core level centered at 530.0 eV was recorded as an internal binding energy reference, and the binding energy span between the O1s and V2p3/2 core level was utilized as a criterion to identify the different vanadium oxidation states according to Silversmit’s method [22]. What is more, the slightly asymmetric feature of the V2p3/2 peak suggests the existence of a certain amount of V^5+^ with a binding energy of 517.4 eV present in the VO_2_ film; no other valence state of V was detected. After subtraction of the Shirley background using the Gaussian–Lorentzian sum function by XPSPEAK 4.1 software, the V2p3/2 peak could be de-convoluted into two peaks, i.e., V^4+^ and V^5+^ peaks. The amount of V^5+^ was supposed to be derived from surface oxidization either in the annealing process or during storage in air, as a slightly oxygen-rich composition generally exists only on the surfaces (the top several nanometers) [9,10]. It has been confirmed that the MIT characteristics might be seriously degraded by the existence of a small amount of vanadium oxides with different stoichiometric composition rather than VO_2_ [20]. Therefore, the passivation layer would be particularly required for extremely thin VO_2_ film; studies on the stability of VO_2_ films and possible solutions to improve their stability by the passivation layer (SiO_2_ or TiO_2_) are being carried out. Here, the ratio of oxygen to vanadium can be roughly estimated to be 2.23 from the integrated area of IV^4+^ and IV^5+^ by the empirical formula: $\left(2I_{V^{4+}}+2.5I_{V^{5+}}\right)/\left(I_{V^{4+}}+\;I_{V^{5+}}\right)$ [20], indicating that V^5+^ has a significant effect on the phase transition of VO_2_ samples, which was coincident with the speculation mentioned above. It was remarkable that the oxygen enrichment on the surface of the sample did not affect the optical properties of VO_2_ thin films.

## 4. Conclusion 

In brief, relatively flat and pure phase VO_2_ films were grown on quartz glass substrates by MBE, with which the film thicknesses were controlled on the atomic scale. A reversible MIT phase transition behaviors were observed from all samples, while the transition magnitude and curve abruptness of thermal hysteresis loops were different, suggesting that a modification of MIT properties could be achieved through the variation of film thickness. Nevertheless, the oxygen enrichment of the surface of the sample made the V^5+^ increase, which in turn caused the MIT property of VO_2_ films to get worse, according to the XPS results. What is more, the temperature on the transmittance of VO_2_ films had a modulation effect, and the IR transmittance of the sample before and after the phase transition is of great variability; and the quartz substrate had no effect on the phase transition characteristics of VO_2_ films. According to the present experimental results, although these samples had poor electrical properties due to the existence of V^5+^, that would not affect their good optical properties, which makes the application of VO_2_ in smart windows for the stoichiometric O/V ratio and crystal quality without high requirements. Such thin thermochromic VO_2_ films are considered to be potentially applicable to smart windows of high total energy efficiency in architecture or automobiles.

## Figures and Tables

**Table 1:** Detailed metal-to-insulator transition (MIT) parameters of the VO_2_/glass samples investigated by the resistance-temperature curve.

Samples	T_c_/°C	ΔH/°C	ΔT/°C	Amplitude
S1	57.0 (Heating)	11.8	16 (Heating)	100
	45.2 (Cooling)		9 (Cooling)	
S2	56.8 (Heating)	7.8	9.3 (Heating)	300
	49.0 (Cooling)		10.3 (Cooling)	
S3	55.3 (Heating)	6.3	3.1 (Heating)	400
	49.0 (Cooling)		11.4 (Cooling)	

**Table 2:** Detailed MIT parameters of the optimized VO_2_/glass samples obtained from temperature-dependent IR transmittance.

Parameters	T_c_/°C	ΔH/°C	ΔT/°C	Amplitude
S2-2 μm	58 (Heating)	4	14 (Heating)	2.6
	54 (Cooling)		13 (Cooling)	
S2-2.5 μm	59 (Heating)	11	11 (Heating)	3.0
	48 (Cooling)		15 (Cooling)	
S3-2 μm	54 (Heating)	3	9 (Heating)	3.5
	51 (Cooling)		13 (Cooling)	
S3-2.5 μm	56 (Heating)	7	14 (Heating)	4.6
	49 (Cooling)		19 (Cooling)	
